# A scoping review on the surgical management of metastatic bone disease of the extremities

**DOI:** 10.1186/s12891-018-2210-8

**Published:** 2018-08-06

**Authors:** Joseph K. Kendal, Annalise Abbott, Sahil Kooner, Herman Johal, Shannon K. T. Puloski, Michael J. Monument

**Affiliations:** 1 Section of Orthopaedic Surgery, Department of Surgery, University of Calgary, 3330 Hospital Drive NW, Calgary, AB T2N 4N1 Canada; 2 McCaig Institute for Bone and Joint Health, University of Calgary, 3280 Hospital Drive NW, Calgary, AB T2N 4Z6 Canada; 3 Centre for Evidence Based Orthopaedics, Departments of Surgery, McMaster University, Hamilton, ON Canada; 4 Department of Health Research Methods, Evidence and Impact, McMaster University, 1280 Main Street West Hamilton, Hamilton, ON L8S 4K1 Canada

**Keywords:** Metastatic bone disease, Orthopedic surgery, Pathologic fracture, Scoping review, Patient-engaged research

## Abstract

**Background:**

Management of metastatic bone disease of the extremities (MBD-E) is challenging, and surgical directions pose significant implications for overall patient morbidity and mortality. Recent literature reviews on the surgical management of MBD-E present a paucity of high-level evidence and global inconsistencies in study design. In order to steer productive research, a scoping review was performed to map and assess critical knowledge gaps.

**Methods:**

The Arksey and O’Malley framework for scoping studies was followed. A comprehensive literature search identified a large body of literature pertaining to the surgical management of MBD-E. Study data and meta-data was extracted and presented using descriptive analytics and a thematic framework. Literature gaps were identified and analyzed.

**Results:**

Three hundred eighty five studies from 1969 to 2017 were included. Studies were categorized into 11 separate themes, with the majority (63%) falling into the “surgical fixation strategies” theme, followed by “complications” at 7% and “prognosis and survival” at 6.2%. Less than 3% of studies were categorized in “patient related outcomes” or “epidemiology” themes. 89% of studies were retrospective and only 6 studies were of level 1 or 2 evidence. We identified a temporal increase in publication by decade, and all studies published on interventional radiology techniques or economic analyses were published after 2007 or 2009, respectively. 64.9% of studies were published in Europe and 20.3% were published in North America. Average patient age was 62 (± 5.2 years), and breast was the most common primary tumour (28%), followed by lung (17%) and kidney (15%). In terms of surgical location, 75% of operations involved the femur, followed by the humerus at 22% and tibia at 3%.

**Conclusions:**

We present a descriptive overview of the current published literature on the surgical management of MBD-E. Critical knowledge gaps have been identified through the development of a thematic framework. Consolidation of literary gaps must involve bolstered efforts towards patient and family-engaged research initiatives and assessment of patient-related surgical outcomes. Multi-disciplinary engagement in developing prospective research will also help guide evidence-based personalized practice for these patients. By building on existing comprehensive patient databases and registries, knowledge on survival and prognostic parameters can be greatly improved.

**Electronic supplementary material:**

The online version of this article (10.1186/s12891-018-2210-8) contains supplementary material, which is available to authorized users.

## Background

Metastatic bone disease is the most common cause for malignant skeletal destruction in adults [1, 2]. As life expectancy in oncology patients continues to improve with advances in imaging, diagnostics, medical treatments and surgical techniques, the health care burden of metastatic bone disease is expected to increase [2–4]. The management of metastatic bone disease is complex and requires the coordination of multidisciplinary teams, including radiation and medical oncologists, orthopaedic surgeons and allied health care professionals.

In patients with metastatic bone disease of the extremities (MBD-E), breast cancer, lung cancer, renal cell cancer, prostate cancer and multiple myeloma contributed to 78% of tumours in the Scandinavian Sarcoma Group (SSG) registry of patients with surgically managed extremity bone metastases [5]. Within the appendicular skeleton, bone metastases most frequently involve the femur, followed by the humerus and tibia [5]. Patients with MBD-E can present with bone pain, ‘impending’ or actual pathologic fracture, or metabolic abnormalities such as hypercalcemia [6]. Options for management of MBD-E include medical interventions (anti-resorptive bone agents, pain medications), palliative radiation therapy and surgical interventions. Surgical management involves stabilization of pathologic bone using various trauma and arthroplasty techniques. Augments and strategies focused on massive bone loss are often needed. The options for operative fixation are varied and dependent on the bony and tumour characteristics including size, location, matrix, and functional deficits. Broadly speaking, prosthesis selection can be categorized into intra-medullary nail fixation, open reduction and internal fixation, and joint reconstruction using conventional arthroplasty or tumour megaprostheses [7]. In some instances bone tumour removal (intralesional curettage, debulking) is required and polymethylmethacrylate (PMMA) cement augmentation is needed for reconstructive construct stability [8]. As patient survival improves with targeted medical therapies, tumour debulking (or perhaps even en bloc resection) and limb reconstruction may continue to play a larger role in the management of oligometastatic extremity bone disease [9–11]. Cement augmentation may also be adjunctively used as a delivery vehicle for antibiotics or cytotoxic tumour drugs [7]. Lastly, minimally invasive image-guided interventional strategies for fixation of MBD-E have been increasingly reported, and include stand-alone percutaneous cement fixation (“cementoplasty”), particularly for patients with short life-expectancies and isolated symptomatic disease foci who are at high risk of complications from surgery [12].

The complexity of managing patients with MBD-E stems from the presence of multiple concurrent factors such as a systemic incurable illness, pathologic bone, and impaired bone and soft tissue healing secondary to previous radiation treatment, malnutrition and immune suppression [7]. Importantly, as these patients are palliative, consideration of patient wishes and immediate quality of life is paramount. This requires a deep awareness of patient medical comorbidities, prognosis and ability to recover from invasive surgery. These unique challenges require surgeons to carefully consider surgical decision-making, fixation strategies and use of adjuncts. As such, management goals in MBD-E are to provide rapid symptomatic relief, optimize mobility and early weight bearing, and to minimize risk of revision surgery [13].

Due to a guarded prognosis and a heterogeneity of disease and practice patterns, coordinated, randomized clinical studies on the surgical management of MBD-E are difficult to perform. A 2014 systematic review on the surgical management of bone metastases identified high rates of peri-operative complications and mortality associated with surgical intervention (17% complication rate, 4% mortality rate), large study heterogeneity in methodology and patient inclusion, and all 45 studies included were of level IV evidence [14]. Another systematic review in 2015 by Janssen et al.*..* assessed the evidence for operative treatment of metastatic humeral fractures [15]. They also identified an overall low level of evidence (six level III and 17 level IV studies included), substantial study heterogeneity in design and intervention, selection bias and poor data reporting on adjuvant treatments and outcome measures such as quality of life, function, and pain. Errani et al also recently performed a systematic review specifically assessing treatment of MBD-E, and included 19 studies; 13 of which were level IV evidence, and the remaining articles were categorized as level III evidence [16]. In general, they identified poor agreement on study conclusions made on management principles, including the utility of en bloc resection, and complication and re-operation rates. Furthermore, the authors generated a treatment algorithm, however due to inconsistent and poor evidence they rely heavily on their large personal expertise and experience to generate their algorithm. Lastly, Willeumier et al. report on a systematic review in 2016 on the use of postoperative radiotherapy for patients with MBD-E. Remarkably, given the widespread use and putative benefits of this adjunctive treatment, they only identified two articles in their review [17]. Furthermore, both of the identified articles were retrospective in nature and of high risk of bias, making it impossible to draw any robust treatment recommendations on the use of postoperative radiotherapy for patients with MBD-E.

There is clearly a paucity of high quality, prospective studies, which is hindering the ability to make evidence-based management decisions in this field. Improving on the current body of literature is vital to advance our knowledge base and to guide appropriate surgical management of this multi-faceted disease process. Furthering our understanding of how to optimize the care of patients with MBD-E will ultimately improve patient outcomes and survival, lead to more efficient utilization of health care resources, and encourage effective multi-disciplinary care.

Scoping reviews are effective strategies to conglomerate and categorize heterogeneous research activity in a large field [18–20]. Our objective was to perform a scoping review of the literature to map research activity in the management of MBD-E in order to accurately define the breadth and depth of the current literature. We hypothesised that the available literature could be thematically organized and analyzed to identify critical knowledge gaps in the literature. Systematic appraisal of literary knowledge gaps in this area is necessary at this time as it will greatly facilitate the development of productive and multifaceted prospective research initiatives within the field of MBD-E.

## Methods

### Project formulation and search strategy

Our research question was initially formulated after our orthopaedic oncology research team identified numerous knowledge gaps in the literature regarding surgical management of MBD-E. A literature search revealed a relative absence of high quality of evidence, so a scoping study methodology was chosen to specifically identify these gaps to more accurately guide future research efforts. Methodology was guided by the Arksey and O’Malley framework for scoping studies [21, 22]. Our comprehensive search strategy was developed with aid of a university librarian to identify articles addressing the issues of managing MBD-E (Additional file 1: Appendix 1). The population of interest includes patients with MBD-E (limited to femur, humerus or tibia) with impending or actual fractures. Clinical studies involving less than 10 patients and literature reviews were excluded from selection. We included all primary research articles, including basic science and biomechanical investigations, economic analyses, prognostic studies, therapeutic studies, surveys and cross-sectional studies published from 1970 to 2017. Relevant articles identified in reference lists were included.

This search strategy was applied to both the Ovid Medline and Embase databases. A pilot search was initially performed and was audited by two orthopaedic oncology surgeons (MM and SP) to achieve consensus on inclusion. Full review was then completed by two reviewers (JK and AA) based on title and abstract screening to generate an article database. 11 themes were identified and agreed upon prior to full data extraction.

### Thematic framework

Fifty articles from the newly generated database underwent full-text review as a pilot study. Preliminary data and meta-data was extracted and reviewed by the research team. Based on this pilot data we generated the thematic framework to categorize articles, and made appropriate adjustments to our extraction form. Overall, we identified 11 literature themes: epidemiology, prognosis and survival, clinical-decision making, surgical fixation strategies, percutaneous image-guided intervention strategies, surgical adjuncts, complications, patient-related outcomes, economic or resource analyses, basic science and miscellaneous (if the article could not be accurately categorized). Thematic selection was determined by the primary objectives of the study and methodology. When studies contained components involving more than one theme, the theme that was most consistently aligned with the research objectives was chosen, as decided by consensus review.

### Data extraction and analysis

We generated a final data extraction document after our pilot search to chart information from the included studies. Full-text review was completed independently by three reviewers (JK, AA and SK), and any disagreements on categorization was resolved by consensus review. Final data abstraction included date of publication, level of evidence, study design, patient demographics, primary tumour diagnosis, treatment strategies, survival data and geographical distribution of research activity. Extracted patient information (primary tumour, intervention, age and survival) was only included if it was not pooled with patients with primary bone tumours or other disease processes. Level of evidence was assessed using the Clinical Orthopaedics and Related Research level of evidence chart, which is an adaptation from published information from the Centre for Evidence-Based Medicine (Oxford, UK) [23]. Descriptive statistics were utilized to assess, tabulate and chart the extracted data.

## Results

### Search results

**Fig. 1:**
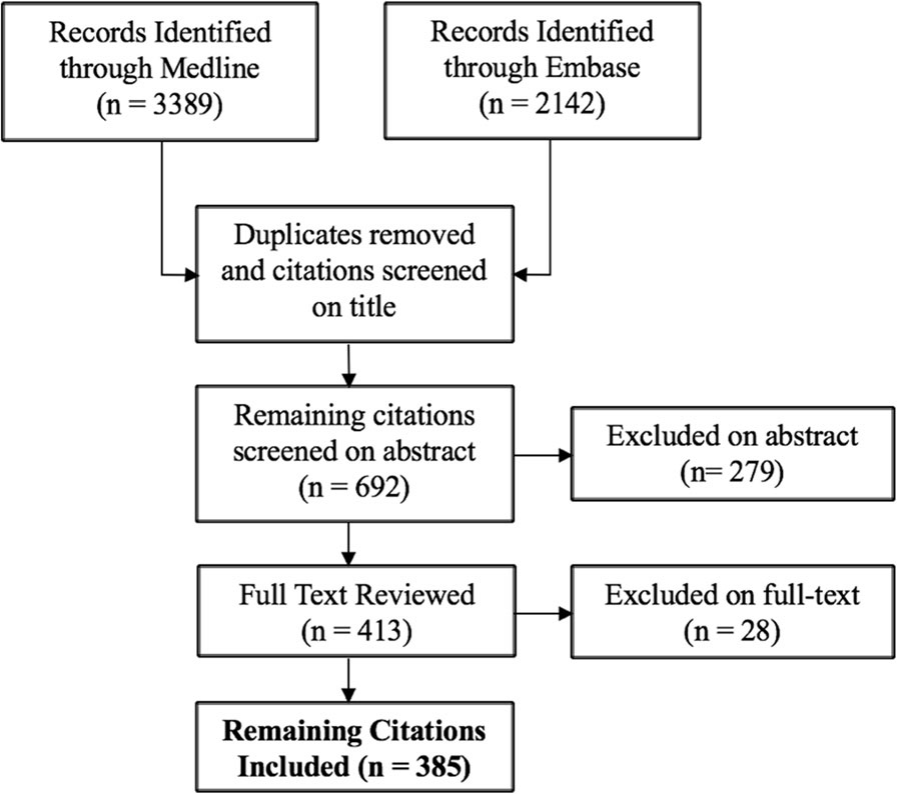
Flow diagram of the article selection process

Our search strategy yielded 3389 articles from Medline and 2142 articles from Embase for screening from 1970 to 2017. Duplicates were removed and the subsequent titles were screened, resulting in 692 remaining articles. Abstracts were reviewed, resulting in a further exclusion of 279 titles. After full-text review, 28 titles were excluded, resulting in 385 articles remaining for data abstraction (Fig. 1).

### Thematic framework

**Fig. 2:**
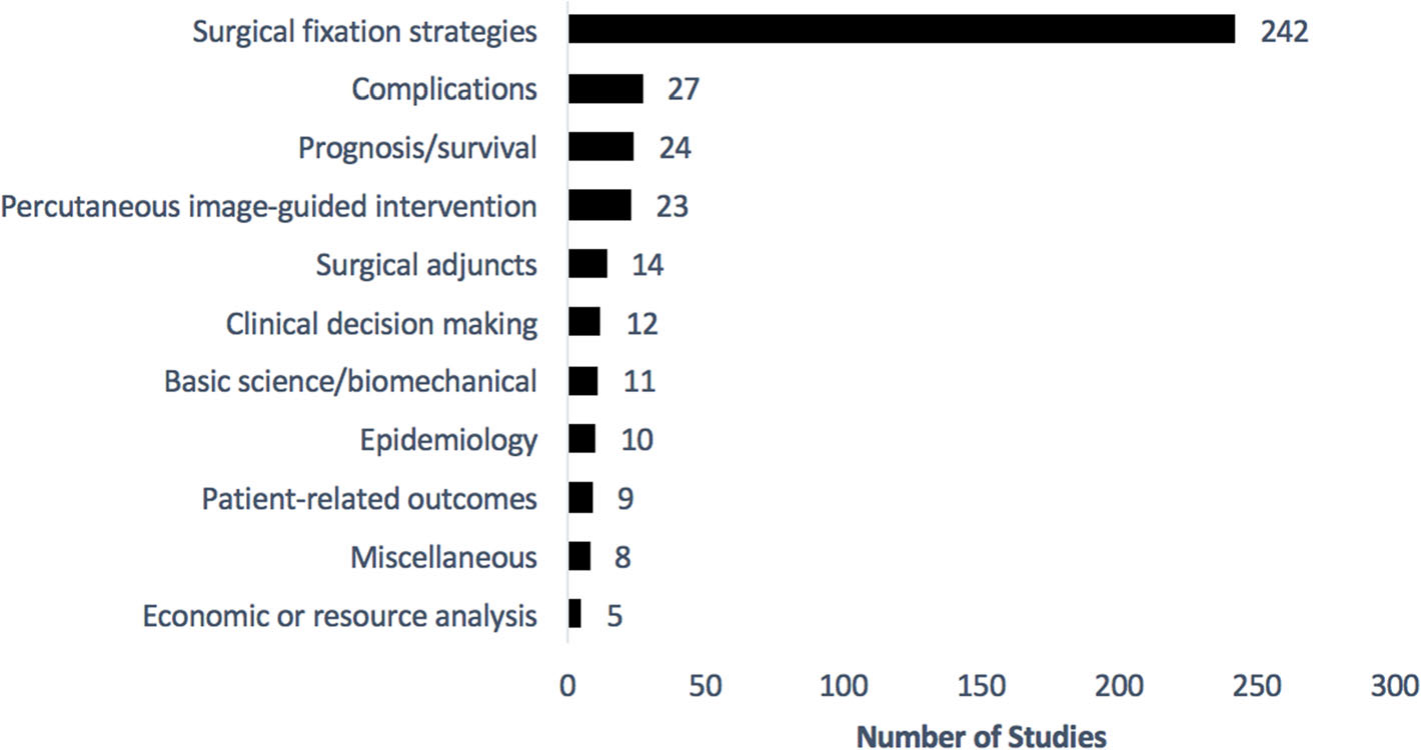
Research Distribution by Study Theme

The majority of the included studies were categorized into the “surgical fixation strategies” theme (63%). These studies were primarily focused on determining the optimal mode of management for different clinical presentations of MBD-E and primarily consisted of case series’. The next most common theme was the “complications” theme at 7%, followed by “prognosis and survival” at 6.2% and “percutaneous image-guided intervention strategies” at 6%. The remaining seven themes each contributed < 5% of the included studies (Fig. 2). These include “surgical adjuncts” (such as radiotherapy and tumour embolization) at 3.6%, “clinical decision making” at 3.1%, “basic science and biomechanical” at 2.9%, “epidemiology” at 2.6%, “patient-related outcomes” at 2.3% and “economic analysis” at 1.3%. The miscellaneous themes accounted for 2.1% of the included studies. While only 6.2% of studies had a primary objective of assessing prognosis and survival in patients with MBD-E, 121 (31.4%) provided extractable survival data for patients with MBD-E.

### Study demographics

**Fig. 3:**
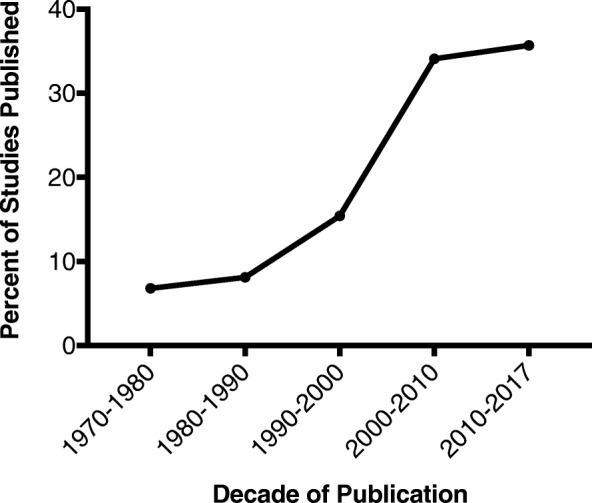
Temporal distribution of publication

Articles were published from a range of 1970–2017, with a median publication year of 2005. Articles published per decade increased with each sequential decade, with a 2.2 times increase in papers published from 2000 to 10 compared to 1990–2000 (Fig. 3). From 2010 to 17, 137 articles have been published, comprising 35.6% of all articles included.

Articles were published in a total of 13 different languages: Chinese, Czech, Danish, Dutch, English, French, German, Italian, Polish, Portuguese, Russian, Spanish and Turkish. The majority of articles were published in English (71%), followed by German (9%), Polish (5%) and French (5%). The remaining 9 languages each contributed to < 5% of included studies (Table 1).

**Table 1:** Study demographics

Characteristic	Number (%)
Publication Language	
English	275 (71.4)
German	36 (9.4)
Polish	21 (5.3)
French	19 (4.9)
Chinese	12 (3.1)
Italian	7 (1.8)
Spanish	7 (1.8)
Dutch	2 (0.5)
Turkish	2 (0.5)
Czech	1 (0.3)
Danish	1 (0.3)
Portuguese	1 (0.3)
Russian	1 (0.3)
Study Design	
Case series	318 (83)
Comparative	41 (10.6)
Cadaver	6 (1.6)
Economic analysis	5 (1)
Biomechanical and Animal Models	5 (1)
Other	4 (1)
Survey	3 (1)
Cross-Sectional	2 (0.5)
Expert Panel	1 (0.3)
Study Perspective	
Retrospective	341 (89)
Prospective	24 (6)
Unable to classify	20 (5)

**Fig. 4:**
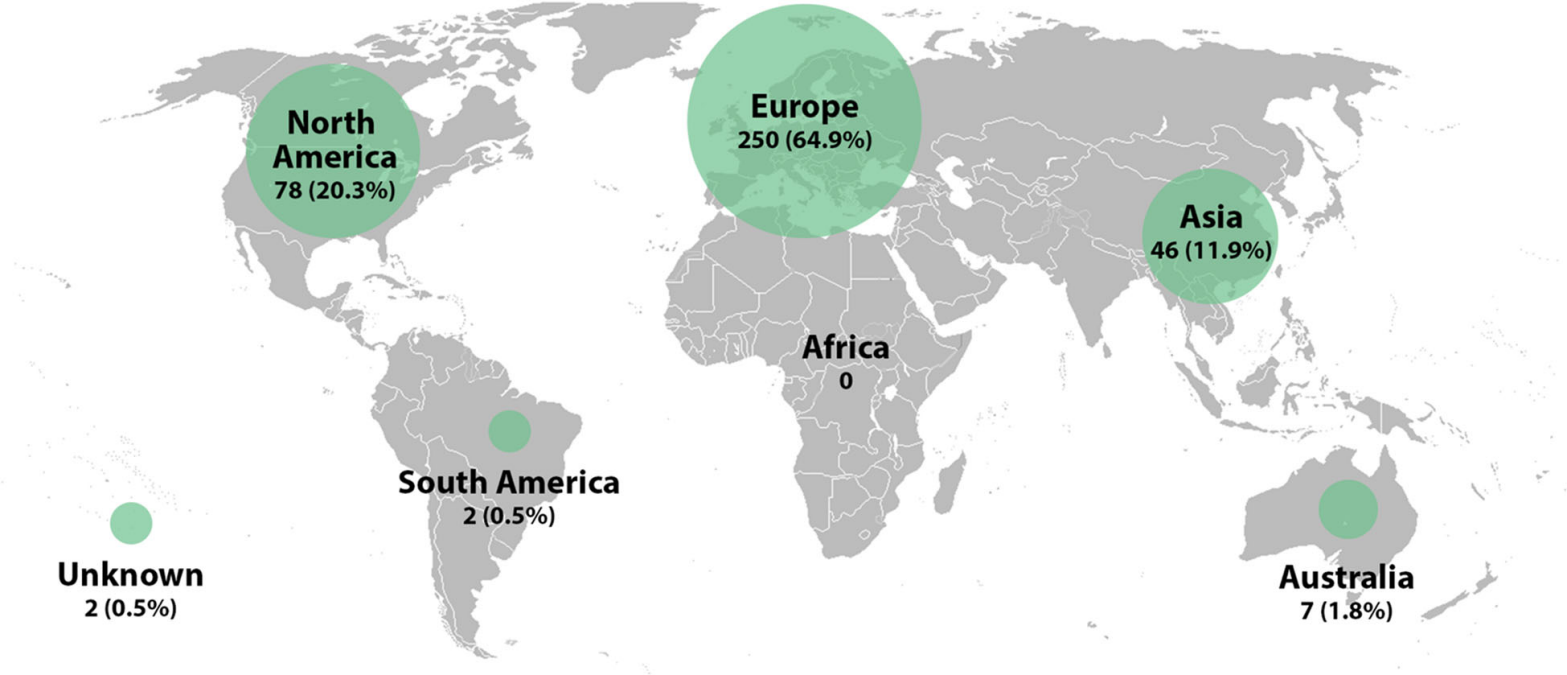
World map representing the geographical distribution of publications. *Map template from ©Cucino/Adobe Stock, Photo #74146873*

Overall, 64.9% of studies were published in Europe, 20.3% published in North America and 12% were published in Asia (Fig. 4). The most common country of publication was USA at 16.9%, followed by Germany at 14.5%. France at 8.1%, Italy at 7.8% and Poland at 7.5%. 2.6% of studies were published in Canada from 1979 to 2012.

### Study design and levels of evidence

**Fig. 5:**
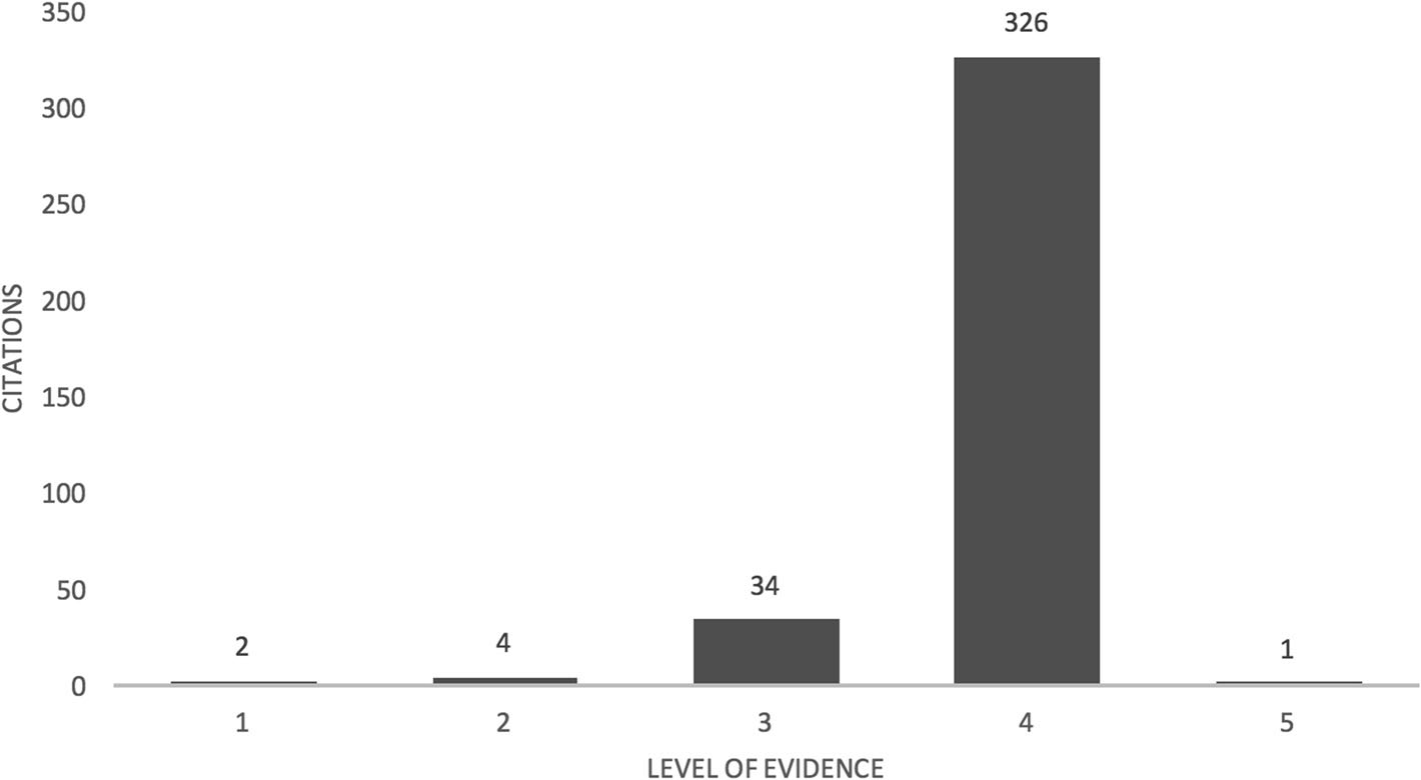
Level of evidence distribution of selected studies

The vast majority of studies were categorized as retrospective (89%), and only 6% of studies identified as prospective (remaining studies were either unable to classify or were not-applicable). 84.7% of articles were level IV evidence, 8.8% were level III, four studies (1%) were level II and two studies were level I (0.5%) (Fig. 5). The most common study design was case series (83%), followed by comparative study (10.6%). The remaining study designs included the following: biomechanical models, cadaver model, cross-sectional study, economic analysis, expert panel, survey, and those that could be classified otherwise were listed as “other” (Table 1). These remaining designs each contributed to ≤2% of total included studies. The two level I studies were both prospective prognostic studies published in 2000 and 2005 and both were categorized under “patient related outcomes.” Of the ten Canadian studies, there were two clinical decision-making surveys (0.5%), one biomechanical analysis (0.3%), six case series’ (1.6%) and one prospective cohort study (0.3%).

### Patient demographics

Mean patient age (included for 34,249 patients) was 62 years with a standard deviation of 5.2 years. Data on surgical or image-guided percutaneous intervention was included for 11,715 cases. The femur was involved in approximately 75% of cases, humerus in 22% and tibia in 3% (Additional file 2: Appendix 2). The most common operation performed was tumour megaprosthesis (38%), followed by intramedullary nail fixation (35%), conventional arthroplasty (16%) and open reduction and internal fixation (10%) (Table 2). For bone specific surgical procedures, the most common intervention was femoral tumour megaprosthesis (29% of total cases), followed by femur intramedullary device fixation (24%). Femur hemi- or total arthroplasty contributed to a further 15% of total cases. Femoral ORIF, cementoplasty and allograft prosthetic composites (APC) accounted for < 5%. Intramedullary device fixation of the humerus comprised 10% of total cases (45% of all humerus cases) followed by humeral tumour megaprosthesis in 6% of all cases. Humeral ORIF, cementoplasty, APC, shoulder arthroplasty and total elbow arthroplasty accounted for < 5% of all cases. Tibial procedures contributed to < 5% and included tumour megaprostheses, intramedullary nails, cementoplasty and ORIF (Additional file 2: Appendix 2). Of 13,167 lesions, the primary tumour diagnosis was distributed as follows: 28% breast, 17% lung, 15% renal, 8% prostate, 7% myeloma, 15% classified as ‘other’, and the remaining primaries each comprising less than 5% (Table 2).

**Table 2:** Patient demographics from included studies

Factors	Number of cases (%)
Primary Tumor	
Breast	3686 (28)
Lung	2277 (17)
Kidney	2002 (15)
Prostate	1079 (8)
Myeloma	928 (7)
Gastrointestinal^a^	381 (3)
Liver	263 (2)
Lymphoma	226 (2)
Thyroid	185 (1)
Melanoma	168 (1)
Plasmacytoma	37 (0.3)
Others	1931 (15)
Anatomic Location	
Femur	8720 (74)
Humerus	2550 (22)
Tibia	370 (3)
Operation method	
Tumour megaprosthesis	4404 (38)
Intramedullary nail	4055 (35)
Conventional arthroplasty	1899 (16)
ORIF	1151 (10)
Cementoplasty	167 (1)
APC	39 (0.3)

See Additional file 2: Appendix 2 for more details on procedures performed

^a^“Gastrointestinal” pooled esophageal, gastric, pancreatic and cholangio-carcinomas

## Discussion

As the health care burden of MBD-E continues to increase, it is critical that we continue to advance our understanding on how to optimally approach the surgical management of these patients [5]. For orthopaedic surgeons, management principles include using an immediately stable implant that provides pain relief and functional preservation for the remainder of a person’s life-span [4]. This requires an understanding of implant performance in a location and lesion-dependent manner. Furthermore, a thorough understanding of factors, such as the patient’s perceived quality of life, expectations, fracture risk and concurrent radiation or chemotherapy is crucial to influence surgical decision making. All of these factors complicating management decision making occur in a climate of rapidly progressing technology and interventional techniques, which provide the treating physician with a myriad of management options. It is clear from multiple recent systematic reviews that a higher quality of research is required to help guide optimal overall treatment of MBD-E [14–17]. Specifically, the current literature reports a paucity of evidence regarding functional outcomes and complications; an abundance of level 3 and 4 evidence; and substantial heterogeneity making it difficult to compare numerous studies.

In an attempt to help steer productive future research, we developed a comprehensive map of research activity on this topic using a scoping review methodology. We included 385 studies and have charted data and meta-data from these studies. As previously identified in systematic reviews on this topic, the level of evidence for included studies was heavily concentrated towards level IV studies. Accordingly, 89% of studies included were retrospective in nature, and the majority (83%) of studies were case series. The second most common study design was a comparative study (10.6%), however, only four of these studies were prospective in nature. These observations are likely influenced by the relatively shorter follow-up period of patients with MBD-E combined with logistical difficulties of performing prospective studies in this patient population.

Our thematic framework identified multiple observable trends. We found a significant concentration of research effort directed towards assessing fixation strategies (63%). Studies assessing interventional radiological strategies were all published after 2007, reflecting a trend towards increased utilization and investigation of this technology. While only 6.2% of studies were categorized in the “prognosis and survival” theme, we recognize that several studies categorized into other themes included survival or prognostic data (31.4% of total studies), however these studies were not focused on survival outcomes or prognosis as a primary study objective. All five studies categorized in the “economic or resource analysis” theme were published after 2009, which may reflect an increasing focus on cost effectiveness and the optimization of health care utilization. With regards to patient-related outcomes, we only identified nine (2.3%) studies that met our criteria (requiring the assessment of patient-related outcomes as a primary objective). These studies included a primary focus on factors such as patient-perceived quality of life, fitness and pain relief, and notably included both of the level I studies identified in this review [24, 25].

The majority of research activity was mapped to Europe (64.9%), with a further 20.3% of articles published from centers in North America (Fig. 4). This finding was consistent with a 2015 scoping review focused on proximal humerus fractures, identifying 64% of research activity in Europe and 21% in North America [19]. Further to these observations is the fact that in our study, the most common country of publications was the USA (16.9%), which was followed by several European countries. These observations may reflect regional concentration of research initiatives and subsequent distribution of multi-center collaborative development. For example, in 1999 the Scandinavian Sarcoma Group (SSG) developed a the world’s largest multi-center prospective registry of surgically treated non-spinal metastases, which was reported to include 1107 patients [5]. Large collaborate registries such as this are critical to improving our data quality and consistency. Notably, no such registry of this magnitude exists in North America. The addition of an analogous North American metastatic bone disease patient registry would substantially enhance our ability to accurately study surgical interventions, prognostication, diagnostics and economic data. Furthermore, this registry would be a source of up to date data with external validity to North American patients.

With respect to surgical interventions, the majority of cases reported were femoral reconstructive procedures. The humerus was the second most common long bone reconstructed, contributing to 22% of cases (of which 45% were fixed with an intramedullary nail). This is consistent with reported frequencies of the humeral metastatic lesions, which account for 16–39% of impending or actual long bone pathologic fractures [26]. In terms of primary malignancy, in this population we also identified breast cancer as the most common long bone metastasizing cancer, followed by lung, renal, and prostate cancers (Table 2). When combined with multiple myeloma, these osteophilic carcinomas contributed to 75% of all cases, which is consistent with reported frequencies from the SSG registry (78%) [5]. These numbers further emphasize considerable footprint of breast carcinoma in the development of MBD-E. For example, it has been shown that the annual incidence of long bone fracture in patients with breast cancer bone metastases is 17%, and a staggering 70% of breast cancer patients have identified bone metastases at the time of death [27, 28].

### Implications for future research activity

Overall, we are observing a steady increase in yearly publication of articles addressing MBD-E (Fig. 3). While this vast amount of new information is contributing to our knowledge of the management of this disease process, we have identified a need to focus our efforts to address a low volume of high quality evidence to guide evidence-based clinical decision making (Table 3). It is recognized that clinical trials in this particular population is difficult; however, previous prospective trials have defined some of these challenges and potential strategies to mitigate them [24, 25]. For example, due to the heterogeneity in clinical presentation, MBD-E cannot be approached as a single disease entity with a predictable time-course and outcome. Accordingly, a large multi-center prospective trial would be necessary in order to detect critical sub-group differences that may help guide who would benefit from aggressive surgical intervention [24]. As previously discussed, the development of a large North American prospective database, such as the SSG registry, is another effective strategy to achieve the patient numbers required to properly study this complex condition.

**Table 3:** Highlighted gaps in the research field identified by the scoping review, with associated research initiatives

Research Gaps	Future Research Initiatives
• Studies focused on patient-related outcomes, such as perceived quality of life indicators • Substantial lack of prospective comparative trials • Knowledge regarding the prognosis and survival of patients with MBD-E, especially in those who undergo a surgical intervention, including en bloc resection • Knowledge regarding survival outcomes of MBD-E patients in the setting of new targeted biologic therapies • Understanding of the health resource and economic implications of MBD-E and its surgical management • Lack of evidence based multi-disciplinary clinical decision-making approaches and algorithms, as well as for surgical adjuncts such as radiotherapy	• Patient and family surveys, multi-disciplinary meetings and trial planning to assess identified knowledge gaps and patient-related outcomes • Development and validation of a patient-related outcome measurement tool for MBD-E • Further development of prospective, multi-centered registries for patients with MBD-E • Further research into economic implications of MBD-E and associated surgical interventions • Assessing the utility of en bloc resection vs. stabilization in oligometastatic disease [31]

With regards to area of research, we identified a large amount of research activity directed towards assessing appropriate fixation strategies, primarily in the form of retrospective case series’. However, there is a stark paucity of research productivity that is primarily focused on assessing patient-related outcomes. This finding is concerning, as the surgical management of MBD-E is generally palliative, with an ultimate primary goal of improving the patient’s remaining quality of life. Two prospective prognostic studies have delineated methodology for assessing patient-related outcome in this population, and have identified methodological areas of weakness as well. For example, the general health status of this patient population is poor at baseline, which contributes to a so-called “floor effect” of outcome scores whereby initial values are too low to detect any further deterioration [24]. Further research efforts into the development of an appropriate, validated outcome tool specific for patients with MBD-E is warranted. This could be further guided by patient and family centered surveys to determine individual perceived benefits and harms of undergoing surgical intervention, and the patient-perceived utility of undergoing adjunctive treatments (ex. tumour debulking) as well. Furthermore, given the importance of understanding patient survival and prognosis, there is a relative lack of high quality research initiatives in this area. Improving on our poor understanding of patient prognostic indicators would provide valuable information to the treating surgeon and patient when making a decision of whether or not to operate, and which procedure to perform. In terms of approaches to treatment, we observed a lack of evidence-based, multi-disciplinary pathways and algorithms to guide care of patients with MBD-E, which is further supported by a recent systematic review [16]. Other future research initiatives must involve assessment of appropriate use of allied health resources such as physiotherapy, occupational therapy and social work. Lastly, surgical adjuncts (including therapies such as radiotherapy and tumour embolization) only encompassed 3.6% of all categorized papers. Given the widespread clinical use of these interventions there is a substantial lack of evidence to inform their appropriate use (including important parameters such as timing of use) [17].

The development of appropriate evidence-based surgical treatment pathways necessitates the consideration of the use of advanced targeted biologic therapies, and prognostic factors such as the primary tumour diagnosis (including sub-type), and metastatic pattern (i.e solitary vs. multiple lesions) [9, 29]. For example, in patients with MBD-E secondary to melanoma, tumour debulking (intralesional curettage or en bloc resection) has been associated with a significantly lower risk of local disease progression, and should be considered as a therapeutic adjunct for these patients [30]. Furthermore, a recent retrospective review presented data indicating that patients with femoral metastases secondary to a breast or kidney primary who responded to targeted biologic therapy have an increased overall survival, but may be at risk of progression of skeletal metastases despite regression of visceral metastases [11]. This may have implications on surgical decision making, as a sarcoma-style tumour resection and limb reconstruction may be beneficial in certain patient populations in order to reduce both tumour burden and the risks of mechanical failure for those surviving > 12 months. This approach may play a particular role in patients with patients with MBD-E secondary to renal cell cancer with oligometastatic disease, as this patient population has been reported to have a 35% 5-year survival rate [29]. In general, more aggressive surgical strategies for patients with MBD-E who are responders to targeted, biologic therapies warrants further consideration and prospective evaluation. Accordingly, through a modified Delphi approach a recent study identified the prospective assessment of the utility of en bloc resection vs. stabilization alone in oligometastatic disease as one of the top four research priorities in all of Orthopaedic oncology [31].

### Limitations

A limitation of our thematic framework included a difficulty in categorizing studies that contained components belonging to multiple themes. In particular, this effect would have over-estimated studies allocated to the “fixation strategies” theme, and a subsequent under-estimation of studies in the “prognosis and survival”, “complications”, “patient-related outcomes” and “surgical adjuncts” themes. However, by using this strict inclusion for the latter four themes, this ensured included articles were specifically related to that category. Furthermore, a limitation to the scoping review methodology is that it is primarily descriptive in nature, and therefore quantitative data analysis was out of the scope of this study. Finally, multiple studies contained pooled data involving primary and bone tumours, and anatomic sites outside of the long bones. For these papers, primary tumour, patient number and age, and interventions had to be excluded.

## Conclusions

We present a comprehensive descriptive overview of the published literature pertaining to the surgical management of MBD-E. Through a thematic framework, we were able to identify critical knowledge gaps that will help direct future research activity. Research priorities for the surgical management of MBD-E include more patient and family-engaged initiatives and improvement in assessment of patient-related outcomes from surgery; further development of databases and registries to help improve understanding on survival and prognosis, and the multi-disciplinary development of prospective clinical trials to bolster the quality of published research.

## Additional files

Additional file 1: **Appendix 1.** Detailed Search Strategies. (DOCX 13 kb)

Additional file 2: **Appendix 2.** Detailed distribution of procedures performed. (DOCX 13 kb)

## Abbreviations

APC: Allograft-Prosthetic Composite
MBD-E: Metastatic Bone Disease of the Extremities
ORIF: Open Reduction and Internal Fixation
PMMA: Polymethylmethacrylate
SSG: Scandinavian Sarcoma Group
