# Supplementary material for: A scoping review on the surgical management of metastatic bone disease of the extremities
Source: BMC Musculoskelet Disord. 2018 Aug 6;19:279. doi: 10.1186/s12891-018-2210-8 (PMC6080518; doi:10.1186/s12891-018-2210-8)
Supplement: Supplementary file 1 — Appendix 1. Detailed Search Strategies. (DOCX 13 kb) [file 12891_2018_2210_MOESM1_ESM.docx]

**Additional File 1: Detailed Search Strategies**

Ovid MEDLINE® In-Process & Other Non-Indexed Citations and Ovid MEDLINE ® (1946 to Present)

1. (bone metast* or metastatic bone disease).mp
2. exp Bone neoplasms/su
3. (long bone* or tibia or femur* or humer* or acetabul* or pelvi*).mp
4. 1 or 2
5. 3 and 4
6. exp Fracture Fixation
7. (resect* or fixation or curettage or cement* or arthroplasty or joint replac* or endoprosthe* or surger* or surgical management).mp
8. 6 or 7
9. 5 and 8
10. Remove duplicates from 9

Embase 1974 to Present

1. (bone metast* or metastatic bone disease).mp
2. exp bone metastasis
3. (long bone* or tibia or femur* or humer* or acetabul* or pelvi*).mp
4. 1 or 2
5. 3 and 4
6. exp fracture fixation
7. (resect* or fixation or curettage or cement* or arthroplasty or joint replac* or endoprosthe* or surger* or surgical management).mp
8. 6 or 7
9. 5 and 8
10. Remove duplicates from 9
