# Supplementary material for: A scoping review on the surgical management of metastatic bone disease of the extremities
Source: BMC Musculoskelet Disord. 2018 Aug 6;19:279. doi: 10.1186/s12891-018-2210-8 (PMC6080518; doi:10.1186/s12891-018-2210-8)
Supplement: Supplementary file 2 — Appendix 2. Detailed distribution of procedures performed. (DOCX 13 kb) [file 12891_2018_2210_MOESM2_ESM.docx]

**Additional File 2. Detailed distribution of procedures performed**

| **Procedure** | **Number (%)** |
| --- | --- |
| *Femur* |  |
| Tumour megaprosthesis | 3356 (28.7) |
| Intramedullary nail | 2825 (24.1) |
| Hemi- or Total Arthroplasty | 1709 (14.6) |
| ORIF | 740 (6.3) |
| Cementoplasty  APC | 66 (0.6)  24 (0.2) |
| *Humerus* |  |
| Intramedullary nail  Tumour megaprosthesis | 1184 (10.1)  755 (6.4) |
| ORIF  Total elbow arthroplasty  Cementoplasty  Shoulder arthroplasty | 402 (3.4)  115 (1)  79 (0.7)  75 (0.6) |
| APC | 15 (0.1) |
| *Tibia* |  |
| Tumour megaprosthesis | 293 (2.5) |
| Intramedullary nail  Cementoplasty | 46 (0.4)  22 (0.2) |
| ORIF | 9 (0.1) |
| APC | 0 |
